# Changes in human skin composition due to intrinsic aging: a histologic and morphometric study

**DOI:** 10.1007/s00418-024-02305-w

**Published:** 2024-07-02

**Authors:** Marta Arnal-Forné, Tamara Molina-García, María Ortega, Víctor Marcos-Garcés, Pilar Molina, Antonio Ferrández-Izquierdo, Pilar Sepulveda, Vicente Bodí, César Ríos-Navarro, Amparo Ruiz-Saurí

**Affiliations:** 1 Department of Pathology, University of Valencia, Avda. Blasco Ibáñez 15. 46010, Valencia, Spain; 2 Instituto de Investigación Sanitaria INCLIVA Biomedical Research Institute, Avda. Menéndez Pelayo 4acc, 46010 Valencia, Spain; 3 Cardiology Department, Hospital Clínico Universitario, Valencia, Spain; 4 Department of Pathology, Instituto de Medicina Legal y Ciencias Forenses, Valencia, Spain; 5 Anatomic Pathology Department, Hospital Clínico Universitario, Valencia, Spain; 6 Centro de Investigación Biomédica en Red (CIBER)-CV, Madrid, Spain; 7 Regenerative Medicine and Heart Transplantation Unit, Instituto de Investigación Sanitaria La Fe, Valencia, Spain; 8 Department of Medicine, University of Valencia, Valencia, Spain

**Keywords:** Skin aging, Intrinsic aging, Morphometric analysis, Human biopsies

## Abstract

Skin represents the main barrier against the external environment, but also plays a role in human relations, as one of the prime determinants of beauty, resulting in a high consumer demand for skincare-related pharmaceutical products. Given the importance of skin aging in both medical and social spheres, the present research aims to characterize microscopic changes in human skin composition due to intrinsic aging (as opposed to aging influenced by external factors) via histological analysis of a photoprotected body region. Samples from 25 autopsies were taken from the periumbilical area and classified into four age groups: group 1 (0–12 years), group 2 (13–25 years), group 3 (26–54 years), and group 4 (≥ 55 years). Different traditional histological (hematoxylin–eosin, Masson’s trichrome, orcein, toluidine, Alcian blue, and Feulgen reaction) and immunohistochemical (CK20, CD1a, Ki67, and CD31) stains were performed. A total of 1879 images photographed with a Leica DM3000 optical microscope were morphometrically analyzed using Image ProPlus 7.0 for further statistical analysis with GraphPad 9.0. Our results showed a reduction in epidermis thickness, interdigitation and mitotic indexes, while melanocyte count was raised. Papillary but not reticular dermis showed increased thickness with aging. Specifically, in the papillary layer mast cells and glycosaminoglycans were expanded, whereas the reticular dermis displayed a diminution in glycosaminoglycans and elastic fibers. Moreover, total cellularity and vascularization of both dermises were diminished with aging. This morphometric analysis of photoprotected areas reveals that intrinsic aging significantly influences human skin composition. This study paves the way for further research into the molecular basis underpinning these alterations, and into potential antiaging strategies.

## Introduction

Skin is the largest human organ and constitutes the main protective barrier against the external environment (Kanitakis 2002), not only in mechanical terms but also thanks to its physicochemical characteristics and to microbiota (Sanford and Gallo 2013). This barrier protects against dehydration, mechanical damage, and biological and physical agents. It also exerts other vital functions, such as thermoregulation, secretion of waste products through sweat, immunological and endocrine functions, as well as its sensory function.

Apart from the roles mentioned above, skin also factors in human relationships as one of the main determinants of beauty (Fink et al. 2001; Sakano et al. 2021), thus influencing social interactions. Products that claim to improve skin tone, glow, and texture, as well as achieving a homogeneous appearance (Sun et al. 2022) are in great demand from the pharmaceutical/cosmetics industry (Baumann 2018). For this reason, knowledge on different levels about skin aging is of special interest, not only at a medical level but also at a social one.

The skin is histologically organized into three layers (Khavkin and Ellis 2011): epidermis, dermis, and hypodermis, within which we find structures known as skin appendages. The epidermis is a multistratified epithelium comprising mainly keratinocytes, but also melanocytes, Merkel cells, and Langerhans cells. The dermis is a connective tissue, made up of cells, fibers, and ground substance, and unlike the epidermis also contains vascular and nervous plexuses. The dermis is divided into papillary (superficial) dermis, which forms the dermal papillae, and reticular or deep dermis, where the fibers (both collagen and elastic) are thicker and horizontally organized, and the deepest part of the skin appendages is found.

Skin aging is manifested by a series of well-known clinical characteristics such as atrophy, wrinkles, rough texture, laxity or loss of elasticity, depigmentation, vascular ectasias, and neoplasms (Zhang and Duan 2018; Pezzini et al. 2023). Two mechanisms of skin aging have been described: extrinsic aging is induced by external factors, including ultraviolet radiation (Berneburg et al. 2000), while intrinsic or chronological aging is regulated by cellular senescence (Csekes and Račková, 2021), oxidative stress (Fisher et al. 2002), and the role of some metalloproteinases, among others.

Since skin aging is an important factor in not only the medical but also the social sphere, the objective of the present study is to morphometrically characterize microscopic changes in human skin composition due to chronological aging. Specifically, alterations in human skin elements due to intrinsic aging are demonstrated via comprehensive histological analysis of human samples isolated from the periumbilical area, a photoprotected body region.

## Materials and methods

### Sample size and study population

This study conforms to the principles for use of human subjects outlined in the Declaration of Helsinki, and the study protocol was approved by the local research ethics committee.

Abdominal skin samples were obtained from the autopsy of 25 patients, who were classified into one of four groups by age: group 1 (0–12 years; *n* = 5), group 2 (13–25 years; *n* = 5), group 3 (26–54 years; *n* = 10), and group 4 (≥ 55 years; *n* = 5). Additional information on case numbers, age, and sex is specified in Table 1.

**Table 1:** Age and sex data of study patients

Case number	Age (years)	Age group^a^	Sex
1	0.17	Group 1	Male
2	54	Group 3	Male
3	35	Group 3	Female
4	75	Group 4	Female
5	62	Group 4	Male
6	52	Group 3	Male
7	17	Group 2	Male
8	48	Group 3	Male
9	41	Group 3	Male
10	58	Group 4	Female
11	1.6	Group 1	Female
12	34	Group 3	Female
13	13	Group 2	Female
14	36	Group 3	Female
15	40	Group 3	Female
16	49	Group 3	Male
17	2	Group 1	Male
18	40	Group 3	Female
19	21	Group 2	Male
20	25	Group 2	Female
21	17	Group 2	Male
22	79	Group 4	Male
23	68	Group 4	Female
24	0.1	Group 1	Male
25	0.1	Group 1	Male

^a^Group 1 (≤ 12 years); group 2 (13–25 years); group 3 (26–54 years); group 4 (≥ 55 years)

Samples, approximately 2.5 cm long and 2.5 cm wide, were isolated from the periumbilical region adjacent to the midline, chosen for its photoprotected location where aging can be attributed mainly to intrinsic causes. Samples were also examined using hematoxylin–eosin and orcein staining techniques to rule out the presence of solar elastosis (characterized by increased elastic material in the papillary dermis), considered one of the main histological characteristics of photoaging (Hunzelmann et al. 2001).

### Microscopic and immunohistochemical analysis

Human skin samples were fixed in 4% paraformaldehyde for 24 h, and subsequently embedded in paraffin. Sections of 5 μm were obtained from the resulting blocks using a microtome, and afterwards fixed to the double gelatin-coated glass slides by heating at 60 °C in an oven for a minimum of 30 min.

Hematoxylin–eosin stain was utilized for histological analysis. To further characterize skin samples, Masson’s trichrome for total collagen, orcein for elastic fibers, Alcian blue counterstained with periodic acid–Schiff staining for coloring mucins in blue, toluidine blue for mast cell granules metachromasia, and Feulgen reaction to reveal cell nuclei were performed. Further details on these protocols are specified in Table 2.

**Table 2:** Reagents used in the histochemical stainings

Stainings	Reagents
Hematoxylin–eosin	Hematoxylin solution (pH 2.7) for 5 min Eosin Y solution 1% in distilled water (pH 4.5) for 4 min
Masson’s trichrome	Harris hematoxylin solution (Ref. 253949, ITW Reagents Panreac) for 5 min Acid fuchsin 1% in distilled water for 5 min Aniline blue 2.5 g in 2 ml acetic acid glacial and 100 ml distilled water for 5 min
Orcein	Orcein 1% in distilled water for 30 min
Alcian blue counterstained with periodic acid–Schiff staining	Alcian blue 1% in 3% acetic acid solution (diluted in distilled water and glacial acetic acid) (pH 2.5) for 30 min Periodic acid PAS 1% in distilled water for 15 min Schiff reagent (Ref. 1.09033.0500, Sigma-Aldrich) for 30 min (in darkness)
Toluidine blue	Toluidine blue 0.01% in distilled water for 15 min
Feulgen reaction	Hydrochloric acid 5% in distilled water for 30 min Schiff reagent (Ref. 1.09033.0500, Sigma-Aldrich) for 45 min (in darkness)

Immunohistochemical stains using specific antibodies were employed to evaluate cell proliferation (anti-Ki67), Merkel cells (anti-CK20), Langerhans cells (anti-CD1a), and blood vessels (anti-CD31). Briefly, sections were firstly subjected to different antigen retrieval methods (Table 3). For heat-induced epitope retrieval, sections were incubated with citrate buffer pH 6.0 or Tris/EDTA buffer pH 9.0 (Dako, Glostrup, Denmark) at 121 ℃ for 3 min. After peroxidase inactivation (H_2_O_2_ 0.3%) and blockade with horse serum, sections were incubated overnight (at 4 ℃) with the specific primary antibodies diluted in PBS/BSA 0.1%. Specific labelling was detected with biotin-conjugated goat anti-mouse IgG antibody (1:500 dilution, Dako Glostrup, Denmark). Further information about primary antibodies and antibody concentration is detailed in Table 3.

**Table 3:** Summary of primary antibody (concentration, antigen retrieval method, and reference) and secondary antibody data used in the study

Antigen	Antigen retrieval method	Primary antibody	Antibody concentration	Secondary antibody
Ki67	Citrate buffer pH 6.0 at 121 ℃ for 3 min	Dako (#IR626)	Undiluted	Biotin-conjugated goat anti-mouse IgG antibody
CK20	Tris/EDTA buffer pH 9.0 at 121 ℃ for 3 min	Dako (#IR777)		
CD1a		Dako (#IR069)		
CD31		Dako (#IR610)		

### Morphometric analysis of the images

Five microscopic photographs of representative areas were taken for each case and stain, using a Leica DM3000 (Leica Microsystems, Wetzlar, Germany) optical microscope. Specific information regarding how photographs were taken to quantify the specific parameters in each stain is detailed in Table 4. A total of 1879 images were morphometrically analyzed using Image ProPlus 7.0 software (Media Cybernetics Inc, Rockville, MD) performed in a blinded manner on coded slides.

**Table 4:** Staining techniques with their corresponding target parameters, photograph magnification data, and morphometric analysis method

Staining technique	Magnification	Measured parameter	Measurements (per photograph) (5 images per case)
Hematoxylin–eosin	×20	Epidermis, ridges and papillae thickness (μm)	10 epidermal measurements (excluding regions of ridges and papillae, as well as artifact-affected epidermal areas) Measurements of all ridges and papillae found
	×40	% Epidermal melanocytes	Manual counting of total epidermal cells and melanocyte cells
	×10	Interdigitation index (relationship between both measured data)	2 measurements per image: Curved length following the silhouette of dermoepidermal junction Distance between both ends forming a straight line
	Macro	Skin appendages (appendages/mm^2^)	Manual counting of hair follicles Manual counting of sweat glands Dermal area
Masson’s trichrome	×10	Papillary dermis thickness (μm)	Minimum 10 measurements per photograph
	×4	Reticular dermis thickness (μm)	Minimum 10 measurements per photograph
Feulgen reaction (DNA revealing)	×40	Total epidermal cellularity/area (cells/mm^2^)	Manual counting of all cell nuclei and measurement of the corresponding area
		Total papillary dermis cellularity relative to area (cells/mm^2^)	
		% epidermal basal cells	Manual counting of total epidermal cells and basal layer cells
	×20	Total reticular dermis cellularity relative to area (cells/mm^2^)	Manual counting of all cell nuclei and measurement of the corresponding area
Toluidine blue	×40	Mast cells per mm^2^ of papillary dermis (cells/mm^2^)	It reveals the metachromasia of mast cell granules, turning them violet, thus allowing manual cell counting Papillary dermis area
Orcein	×20	% of area occupied by elastic fibers in reticular dermis	Elastic fibers area Reticular dermis area
Alcian blue	×40	% of area occupied by mucopolysaccharides in papillary and reticular dermis	Mucopolysaccharides area Papillary and reticular dermis areas
Ki67 (cell proliferation marker)	×20	Mitotic index (ratio between cells in mitosis and total epidermal cells)	Manual counting of mitotic cells Manual counting of epidermal cells
CK20	×40	Merkel cells (cells/mm^2^)	Manual counting of Merkel cells Epidermal area
CD1a	×40	Langerhans cells (cells/mm^2^)	Manual counting of Langerhans cells Epidermal area
CD31 (endothelial cell marker)	×10	Skin vascularization (blood vessels/mm^2^)	Manual counting of papillary and reticular dermal blood vessels Papillary and reticular dermis areas

### Statistical analysis

The Kolmogorov–Smirnov normality test was performed for each variable. Variables were expressed as the mean and standard deviation. Unpaired Student *t* tests were used for comparisons and statistical significance was considered for two-tailed *p* value less than 0.05. GraphPad Prism 9.0 (GraphPad Software, Boston, USA) was used throughout.

## Results

### Changes in epidermal and dermal width at different ages

**Fig. 1:**
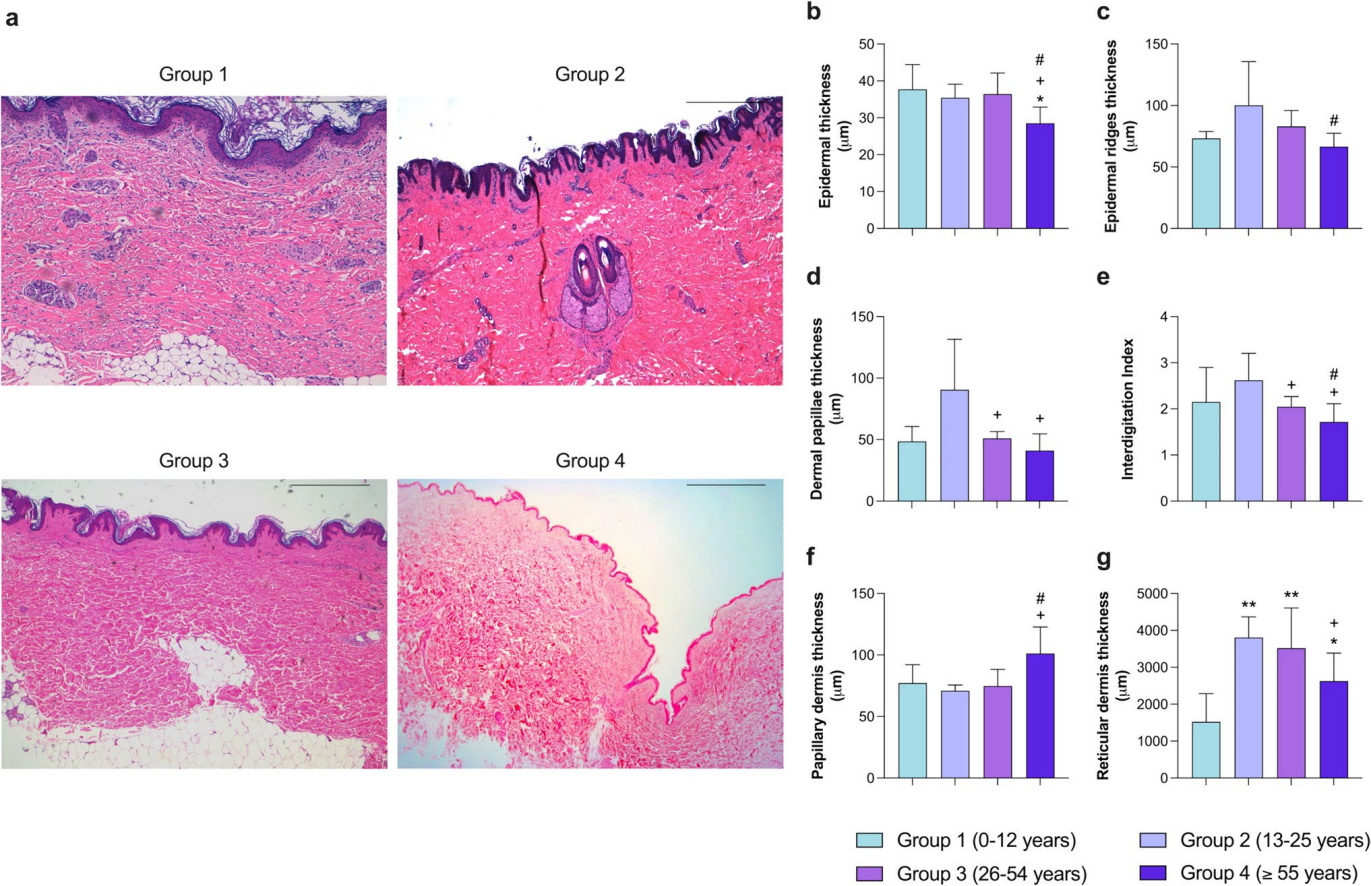
Morphometric comparison of epidermal and dermal thickness at different ages (**a**). Representative macro images of human photoprotected skin samples isolated from patients in group 1 (0–12 years; *n* = 5), group 2 (13–25 years; *n* = 5), group 3 (26–54 years; *n* = 10), and group 4 (≥ 55 years; *n* = 5) stained with hematoxylin–eosin. Morphometric quantification of epidermal thickness (**b**), epidermal ridges thickness (**c**), dermal papillae thickness (**d**), interdigitation index (**e**), papillary dermis thickness (**f**), and reticular dermis thickness (**g**). Images were analyzed with Image-Pro Plus analysis software. Scoring was performed by a blinded observer unaware of the experimental group. Data were analyzed by unpaired Student *t* test. *p < 0.05, **p < 0.01 vs. group 1; +*p* < 0.05 vs. group 2; #*p* < 0.05 vs. group 3. Bar represents 2 mm

To provide a general overview of variations in skin layers and their relationship with aging, quantification of the thickness of the epidermis, epidermal ridges, and dermal papillae were firstly performed. Figure 1a displays representative images taken at lower magnifications from the four groups.

A clear reduction in epidermal thickness was detected in the oldest group compared to group 1 (*p* = 0.01), 2 (*p* < 0.05), and 3 (*p* < 0.05) (Fig. 1b). Epidermal ridge thickness peaks in youth (group 2), and a significant decrease (*p* < 0.05) is seen between groups 3 and 4 (Fig. 1c). A similar trend was observed studying the dermal papillae (Liao et al. 2014), whose length was significantly shorter in groups 3 and 4 compared to group 2 (*p* < 0.05) (Fig. 1d). Furthermore, an almost significant reduction was demonstrated comparing the papillary measurements of people over 55 years old with adults between 26 and 54 years (*p* = 0.08). Finally, a slight although not statistically significant increase was seen in ridge and papillae measurements between group 1 and 2 (*p* = 0.09).

To analyze the stability of the dermoepidermal junction, the interdigitation index, which considers the surface contact between both layers of the skin, was calculated. In comparison to younger adults (group 2), the interdigitation index was significantly reduced (*p* < 0.05) in the 26–54-year-old group and this decay was more pronounced in patients from the oldest group (*p* < 0.05) (Fig. 1e).

Lastly, papillary and reticular dermis thicknesses were measured, revealing a slight increase in papillary dermal thickness with age, statistically significant when comparing group 4 (≥ 55 years) with younger adults (groups 2 and 3) (*p* < 0.05), and almost significant (*p* = 0.07) when comparing it with the youngest age group (≤ 12 years) (Fig. 1f).

In contrast, our study of changes in reticular dermis thickness (Fig. 1g) showed above all an increase after childhood and a posterior regression in old age. This translates into a significant increase in its width in group 2 (*p* < 0.01), group 3 (*p* < 0.01), and group 4 (*p* < 0.05) compared to group 1 (children), being significantly lower in the older group (≥ 55) than in younger people (group 2) (*p* = 0.059). Numerical results are presented in Table 5.

**Table 5:** Numerical results obtained in each measured parameter for this study

	Measured parameter	Mean ± standard deviation			
		Group 1	Group 2	Group 3	Group 4
Macroscopic measures (Fig. 1)	Epidermis thickness (μm)	37.73 ± 6.70	35.43 ± 3.69	36.38 ± 5.72	28.53 ± 4.35
	Papillary dermis thickness (μm)	77.18 ± 14.82	70.95 ± 4.65	74.85 ± 13.50	101.2 ± 21.43
	Reticular dermis thickness (μm)	1521.00 ± 765.70	3806.00 ± 556.50	3521 ± 1087	2627.00 ± 758.70
	Dermal papillae thickness (μm)	48.62 ± 12.13	90.57 ± 41.00	51.09 ± 5.50	41.05 ± 5.59
	Epidermal ridges thickness (μm)	73.37 ± 5.40	100.20 ± 35.56	83.10 ± 12.80	66.6 ± 10.78
	Interdigitation index	2.15 ± 0.75	2.62 ± 0.59	2.05 ± 0.22	1.72 ± 0.39
Epidermis (Fig. 2)	Total cellularity (cells/mm^2^)	14,553 ± 2339	13,175 ± 2589	12,194 ± 2528	12,480 ± 2910
	Basal cells (%)	31.14 ± 3.12	29.52 ± 4.79	28.31 ± 4.53	29.56 ± 3.31
	Melanocytes (cells/mm^2^)	4.52 ± 0.59	8.65 ± 2.29	8.99 ± 3.42	9.93 ± 4.54
	Mitotic index (a.u.)	0.14 ± 0.02	0.13 ± 0.07	0.10 ± 0.03	0.05 ± 0.02
	Merkel cells (cells/mm^2^)	42.95 ± 42.95	7.71 ± 9.48	21.00 ± 23.75	31.10 ± 28.87
	Langerhans cells (cells/mm^2^)	326.60 ± 32.59	384.80 ± 68.12	454.20 ± 108.30	457.40 ± 110.00
Papillary dermis (Fig. 3)	Total cellularity (cells/mm^2^)	4556.00 ± 700.70	3446.00 ± 647.10	3017.00 ± 591.20	2714.00 ± 450.50
	Mast cells (cells/mm^2^)	81.44 ± 27.46	134.10 ± 31.25	94.26 ± 21.30	118.30 ± 42.80
	Glycosaminoglycans (% of area)	17.71 ± 3.34	33.87 ± 2.28	31.50 ± 3.56	25.38 ± 10.59
	Blood vessels (vessels/mm^2^)	438.20 ± 106.40	374.30 ± 98.31	296.30 ± 125.10	203.50 ± 84.28
Reticular dermis (Fig. 4)	Total cellularity (cells/mm^2^)	1462.00 ± 788.80	358.10 ± 48.85	343.00 ± 158.10	246.50 ± 85.72
	Glycosaminoglycans (% of area)	5.02 ± 0.87	9.46 ± 2.39	4.55 ± 1.68	2.49 ± 0.79
	Elastic fibers (% of area)	9.52 ± 4.55	7.69 ± 3.28	5.36 ± 1.26	5.73 ± 2.76
	Blood vessels (vessels/mm^2^)	40.27 ± 6.46	48.89 ± 15.78	40.70 ± 15.33	28.54 ± 10.93
Annexes (Fig. 5)	Hair follicles (number/mm^2^)	0.33 ± 0.18	0.18 ± 0.07	0.08 ± 0.05	0.05 ± 0.02
	Sweat glands (number/mm^2^)	0.48 ± 0.12	0.12 ± 0.09	0.11 ± 0.08	0.12 ± 0.08

Group 1 (0–12 years; *n* = 5), group 2 (13–25 years; *n* = 5), group 3 (26–54 years; *n* = 10), group 4 (≥ 55 years; *n* = 5)

### Effects of intrinsic aging on epidermal cellularity

**Fig. 2:**
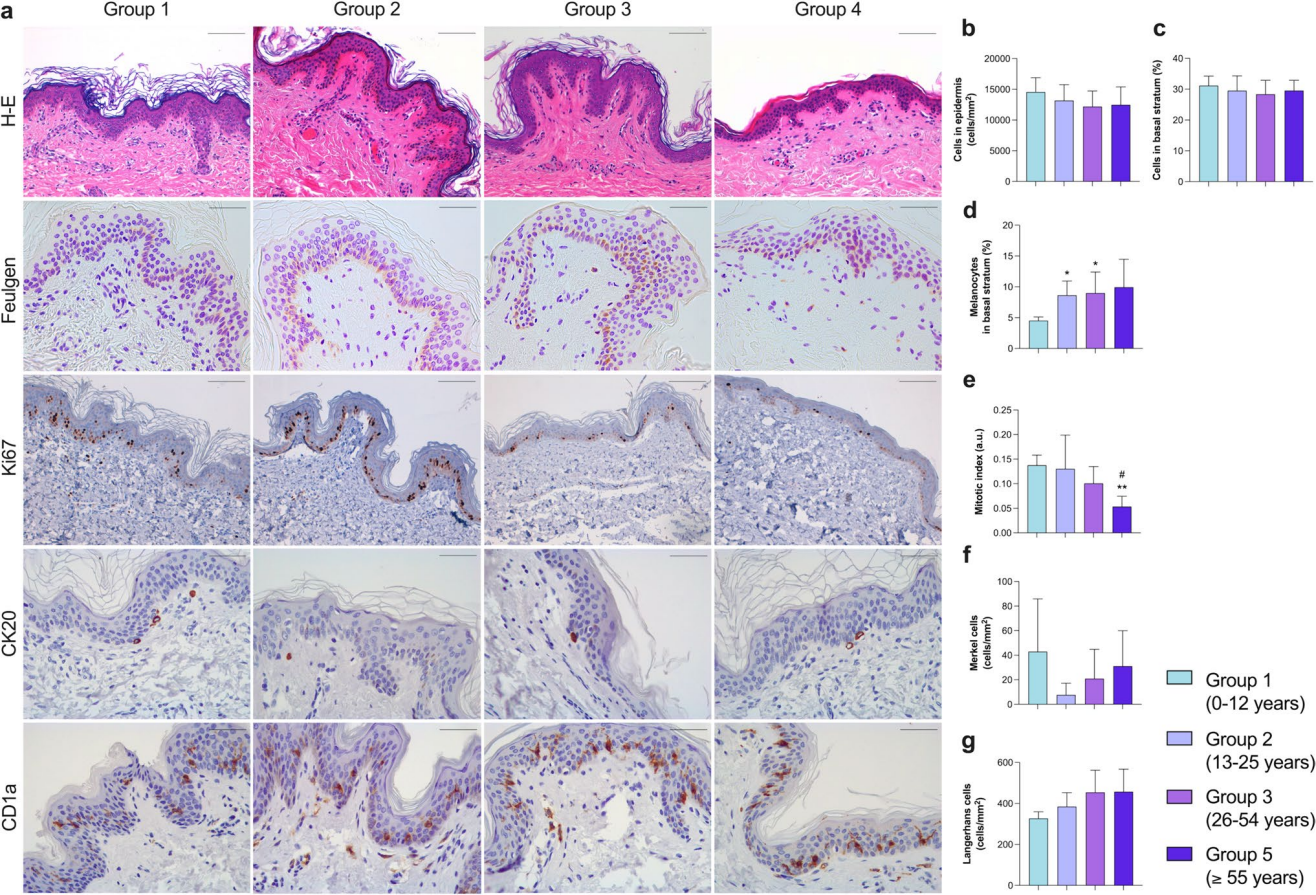
Morphometric changes in epidermal components due to intrinsic aging (**a**). Representative images of human photoprotected skin samples isolated from patients in group 1 (0–12 years; *n* = 5), group 2 (13–25 years; *n* = 5), group 3 (26–54 years; *n* = 10), and group 4 (≥ 55 years;* n* = 5) stained with hematoxylin–eosin (upper panel, ×20), Feulgen technique (marker of total cell nuclei, upper-central panel, ×40), anti-Ki67 (marker of cell proliferation, central panel, ×20), anti-CK20 (marker of Merkel cells, lower-central panel, ×40), and anti-CD1a (marker of Langerhans cells, lower panel, ×40). In the epidermis, morphometric quantification of total cells (**b**), percentage of basal cells (**c**), melanocytes in the basal stratum (**d**), mitotic index (**e**), number of Merkel cells (**f**), and number of Langerhans cells (**g**). Images were analyzed with Image-Pro Plus analysis software. Scoring was performed by a blinded observer unaware of the experimental group. Data were analyzed by unpaired Student *t* test. *p < 0.05, **p < 0.01 vs. group 1; #*p* < 0.05 vs. group 3. Bar represents 100 μm in H-E and Ki67; 50 μm in Feulgen, CK20 and CD1a. *H*-*E* hematoxylin–eosin

After epidermal and dermal width were compared among different age groups, alterations in epidermis composition due to intrinsic aging were analyzed. According to our data, there was a slight decrease in epidermal cellularity (*p* > 0.05) (Fig. 2b). Specifically, the percentage of melanocytes in the basal layer was significantly increased (Fig. 2d), especially comparing group 1 with groups 2 (*p* < 0.05) and 3 (*p* = 0.05), whereas no differences were observed regarding the proportion of basal cells in the epidermis (Fig. 2c).

To further scrutinize the changes in other cell types, the presence of Merkel cells (neuroendocrine cells, type 1 mechanoreceptors involved in touch sensation) and Langerhans cells (antigen-presenting dendritic cells) was determined. Concerning Merkel cells, dynamic changes caused by intrinsic aging were noticed: the number of mechanoreceptors was highest in the early stages of life, with a sharp drop in the 13–25 years group and a partial recovery afterwards (Fig. 2f). Contrariwise, the number of Langerhans cells rose with increasing age (Fig. 2g).

Lastly, the mitotic index, resulting from the ratio between mitotic epidermal cells and total epidermal cellularity, was calculated as a marker of tissue regeneration capacity. A dramatic drop in this index was detected in the oldest group compared to the 0–12 years (*p* < 0.01) and the 26–54 years groups (*p* < 0.05) (Fig. 2e). Numerical results are shown in Table 5.

### Changes in dermal composition with age

**Fig. 3:**
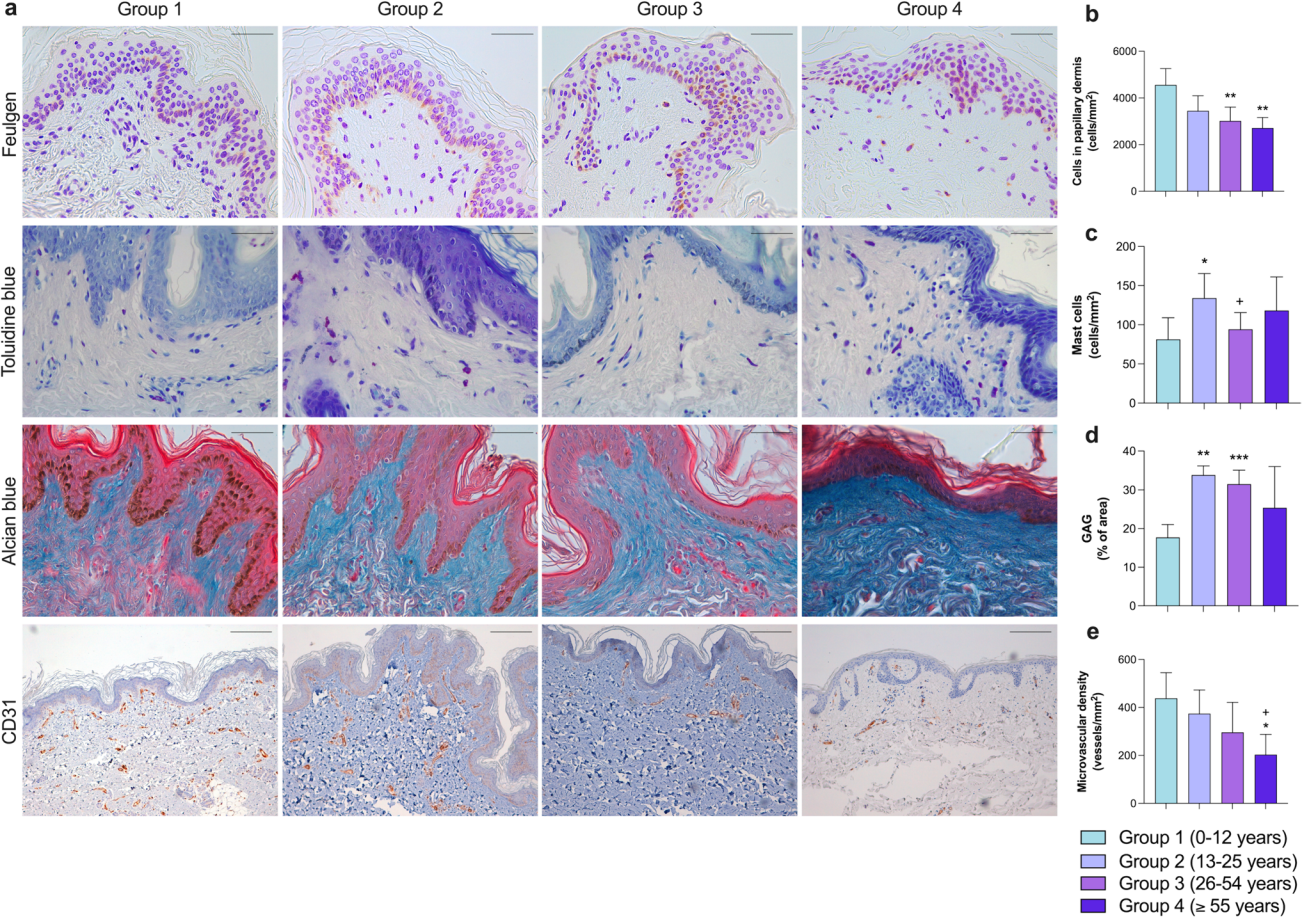
Evaluation of alteration in papillary dermis composition caused by intrinsic aging (**a**) Representative images of human photoprotected skin samples isolated from patients in group 1 (0–12 years; *n* = 5), group 2 (13–25 years; *n* = 5), group 3 (26–54 years; *n* = 10), and group 4 (≥ 55 years; *n* = 5) stained with Feulgen technique (marker of total cell nuclei, upper panel, ×40), toluidine blue (marker of mast cells, upper-central panel, ×40), Alcian blue (marker of GAG, lower-central panel, ×40), and anti-CD31 (marker of endothelial cells, lower panel, ×10). In papillary dermis, morphometric quantification of number of total cells (**b**), number of mast cells (**c**), percentage of area occupied by GAG (**d**), and number of vessels (**e**). Images were analyzed with Image-Pro Plus analysis software. Scoring was performed by a blinded observer unaware of the experimental group. Data were analyzed by unpaired Student *t* test. *p < 0.05, **p < 0.01, ***p < 0.001 vs. group 1; +*p* < 0.05 vs. group 2. Bar represents 50 μm in Feulgen, toluidine blue, and Alcian blue; 200 μm in CD31. *GAG* glycosaminoglycans

**Fig. 4:**
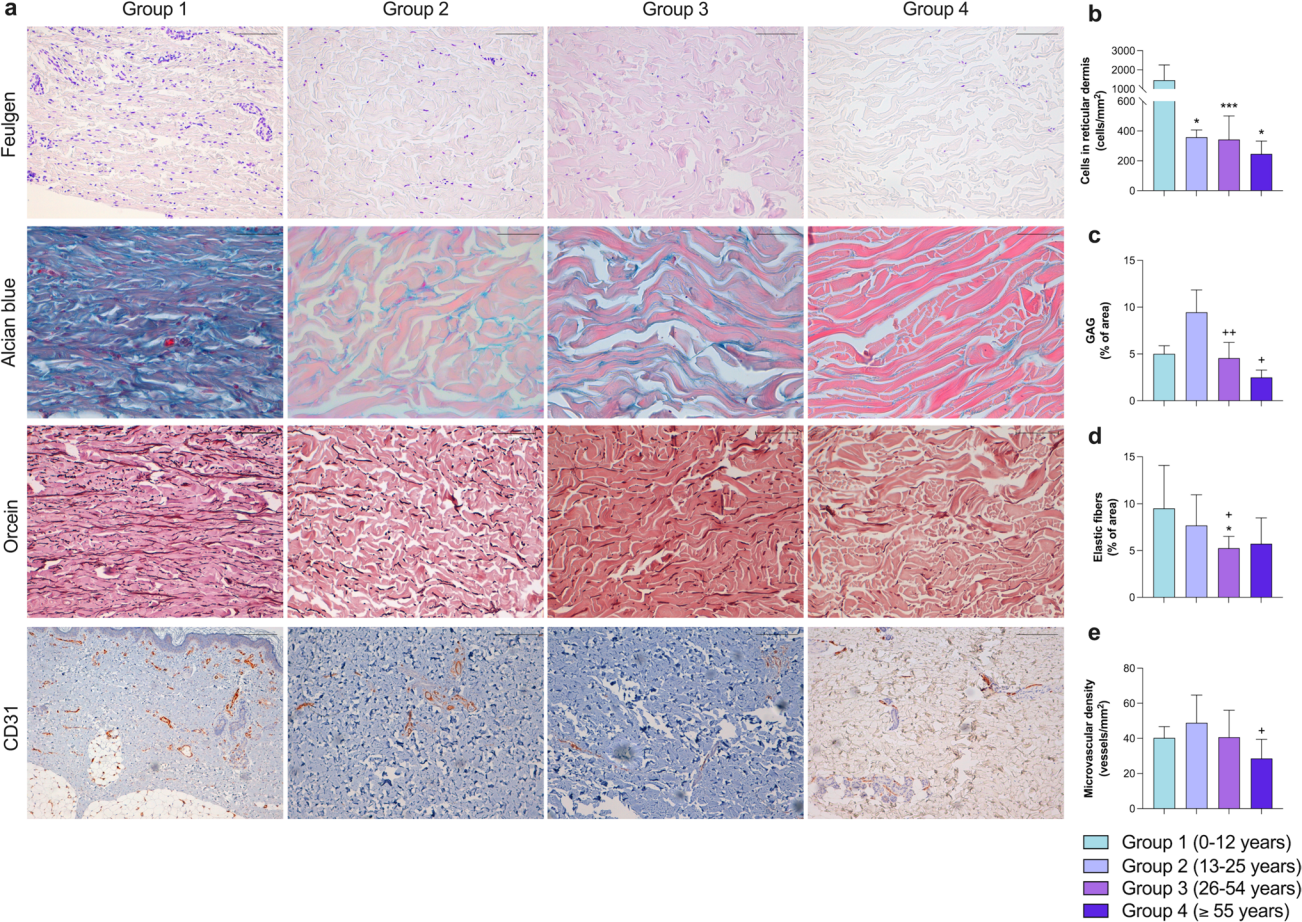
Effect of intrinsic aging on human reticular dermis composition (**a**). Representative images of human photoprotected skin samples isolated from patients in group 1 (0–12 years; *n* = 5), group 2 (13–25 years; *n* = 5), group 3 (26–54 years; *n* = 10), and group 4 (≥ 55 years; *n* = 5) stained with Feulgen technique (marker of total cell nuclei, upper panel, ×20), Alcian blue (marker of GAG, upper-central panel, ×40), orcein staining (marker of elastic fibers, lower-central panel, ×20), and anti-CD31 (marker of endothelial cells, lower panel, ×10). In reticular dermis, morphometric quantification of number of total cells (**b**), percentage of area occupied by GAG (**c**), percentage of area occupied by elastic fibers (**d**), and number of vessels (**e**). Images were analyzed with Image-Pro Plus analysis software. Scoring was performed by a blinded observer unaware of the experimental group. Data were analyzed by unpaired Student *t* test. *p < 0.05, ***p < 0.001 vs. group 1; +*p* < 0.05, ++*p* < 0.01 vs. group 2. Bar represents 100 μm in Feulgen and orcein; 50 μm in Alcian blue; 200 μm in CD31. *GAG* glycosaminoglycans

Morphometric analysis of alterations in different dermal components due to intrinsic aging was also performed. In both papillary and reticular dermis, a gradual reduction in total cell count was observed with aging, exhibiting a significant reduction in the number of cells in 26–54-year-olds and the ≥ 55 group compared to children in group 1 (*p* < 0.01) (Figs. 3b and 4b). In contrast, a clear rise in the number of mast cells (Kritas et al. 2013), white blood cells that reside in connective tissues and play a fundamental role in innate immune defense, was detected inside the papillary dermis in group 2 compared to group 1 (*p* < 0.05), and a subsequent decrease in adults between 26 and 54 years compared to people aged 13–25 years old (*p* < 0.05), while the cell count remained unchanged in group 4 (Fig. 3c).

In terms of extracellular matrix, a significant augmentation in the density of elastic fibers within the reticular dermis was detected in group 3 compared to groups 1 (*p* < 0.05) and 2 (*p* = 0.059), as was a slight although not significant trend towards an increase in old age (Fig. 4d). Regarding glycosaminoglycans (GAG), crucial for water retention and preserving tissue turgor, differing behavior was observed in the two dermal regions. In the papillary dermis (Fig. 3d), the percentage of area occupied by GAG increased significantly (*p* < 0.01) from youth onwards and remained high with advancing age (in groups 3 and 4). In contrast, in the reticular dermis (Fig. 4c), a fall was evidenced in the presence of GAG in group 3 (p < 0.01) and group 4 (*p* < 0.05) compared to the young people from group 2 (Table 5).

Lastly, dermis vascularization (Figs. 3e and 4e) was quantified in both areas as the number of vessels per area, showing significant reduction with aging. In papillary and reticular dermis, the number of microvessels was minimal in the oldest group compared to people between 13 and 25 years old (*p* < 0.05).

### Skin appendages: sweat glands and hair follicles

**Fig. 5:**
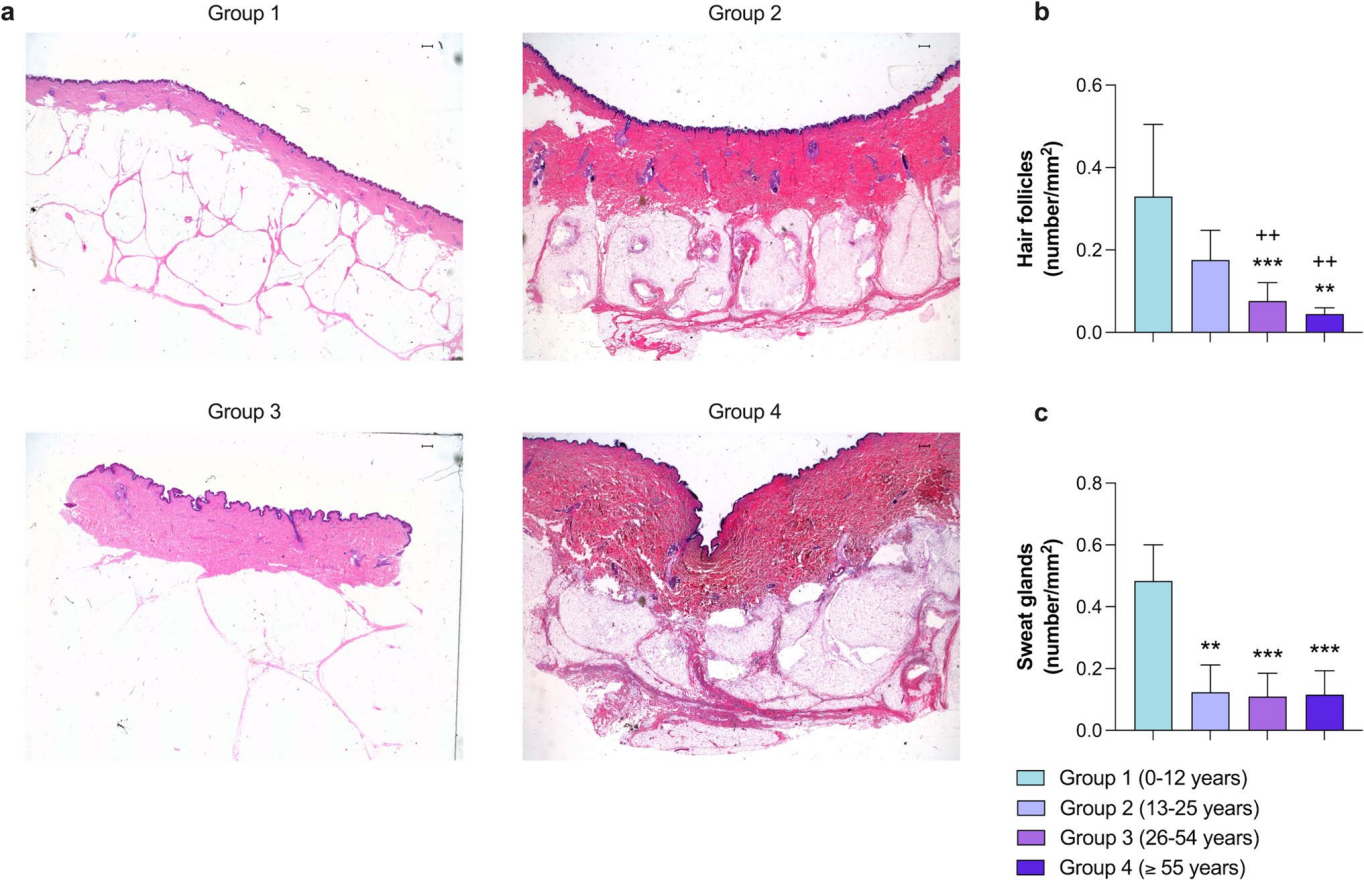
Variations in the number of human skin appendages due to intrinsic aging (**a**). Representative ×1.6 images of human photoprotected skin samples isolated from patients in group 1 (0–12 years; *n* = 5), group 2 (13–25 years; *n* = 5), group 3 (26–54 years; *n* = 10), and group 4 (≥ 55 years; *n* = 5) stained with hematoxylin–eosin. Morphometric quantification of the number of hair follicles (**b**) and sweat glands (**c**). Images were analyzed with Image-Pro Plus analysis software. Scoring was performed by a blinded observer unaware of the experimental group. Data were analyzed by unpaired Student *t* test. **p < 0.01, ***p < 0.001 vs. group 1; ++*p* < 0.01 vs. group 2. Bar represents 500 μm

Both sweat glands and hair follicles reduced in number in the older age groups compared to children (group 1) (Fig. 5). Specifically, significant differences (*p* < 0.001) in the number of hair follicles were found between the children and groups 3 and 4. As regards sweat glands, statistically significant differences (*p* < 0.01) also resulted from comparison between groups 1 and 2. Numerical results are shown in Table 5.

## Discussion

**Fig. 6:**
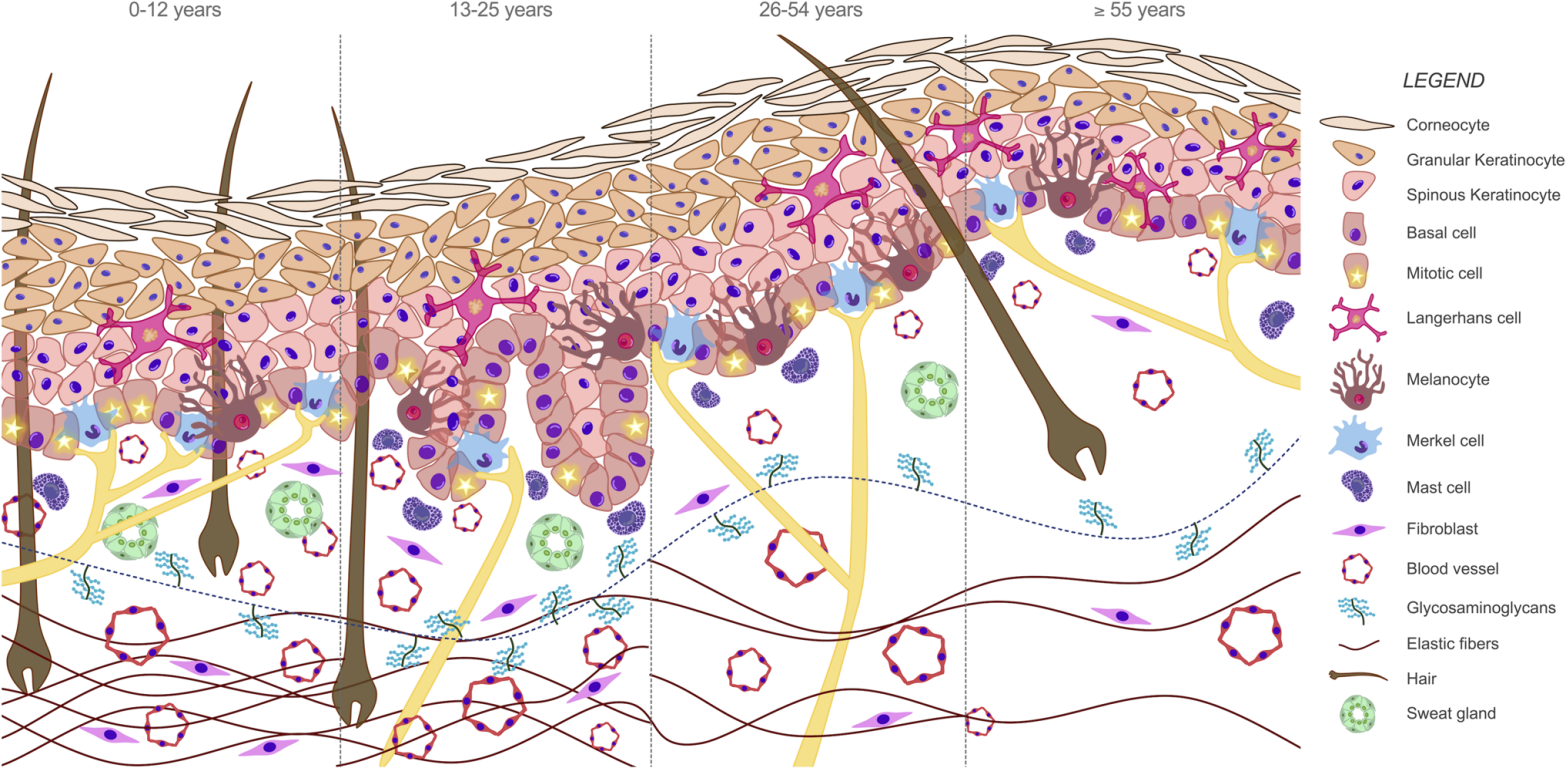
Changes in skin composition due to intrinsic aging. A schematization of skin histological structure evolution in photoprotected regions only affected by chronological aging. This model clearly compares variations in all studied parameters between the age groups: group 1 (0–12 years; *n* = 5), group 2 (13–25 years; *n* = 5), group 3 (26–54 years; *n* = 10), group 4 (≥ 55 years; *n* = 5)

According to our data, intrinsic aging has a dramatic effect on human skin composition as reflected by reduced epidermal thickness, flattened dermo-epidermal junction, and the increase in papillary dermis thickness, probably due to the accumulation of GAG. Additionally, reticular dermis thickness decays with age, mirroring GAG and elastic fibers decline. Lastly, the number of vessels, cells, and appendages is diminished in dermis throughout a person’s lifetime (Fig. 6).

### Physiology and microscopic structure of human skin

Skin is the largest human organ, extending over more than 2 m^2^ of surface and representing about 15% of total body weight (Kanitakis 2002). It provides the principal barrier against dehydration, mechanical damage, and biological (i.e., microorganisms and toxins) and physical agents, including UV radiation and temperature changes. Furthermore, the skin plays an active role in thermoregulation, the secretion of waste products through sweat, as well as in immunological, sensory, and endocrine regulation.

In terms of microscopic structure (Khavkin and Ellis 2011), the skin is stratified into three layers, from superficial to deep: epidermis, dermis, and hypodermis. The epidermis, ectoderm-derived, is the outermost layer of the human body, and its predominant cells are keratinocytes (producers of keratin for skin semipermeability maintenance), but it also contains melanocytes, Merkel cells, and Langerhans cells. The dermis, in contrast, is made up of fibers, ground substance, and cells, and contains vascular and nervous plexuses.

Macroscopic changes in human skin due to aging are easily identifiable (Haydont et al. 2019), including wrinkles, atrophy, irregular pigmentation, and laxity (Zhang and Duan 2018). The aging process is regulated by two different mechanisms: extrinsic and intrinsic aging. The former is induced by external factors, known as the exposome: mainly ultraviolet radiation (Berneburg et al. 2000), although others such as environmental pollution and smoking can also play a role. Conversely, intrinsic or chronological aging is a consequence of cellular senescence (Csekes and Račková, 2021), a reduced capacity for cell proliferation, seen especially in basal cells (Zhang and Duan 2018); oxidative stress (Fisher et al. 2002) (a dysfunction of mitochondrial molecules that leads to the formation of reactive oxygen species), and even the activity of some metalloproteinases.

The present research is focused on examining chronological skin aging, thus leaving aside the effect of external factors such as photoaging, given that skin samples are taken from a photoprotected body region, the periumbilical area using morphometric analysis. This technique has been previously employed to determine the microscopic changes in skin parameters due to different interventions (Costello et al., 2023; Demyashkin et al. 2023; Gogly et al. 1997).

### Morphometric changes in epidermal components due to intrinsic aging

Turning to changes in epidermal thickness due to intrinsic aging, a decrease over age, especially notable in the oldest group, was detected. Firstly, one might think that this reduction could be due to a drop in the number of epidermal cells. To corroborate this hypothesis, total epidermal cellularity and mitotic index (reflecting the relationship between dividing and total cells) were morphometrically assessed. In terms of the mitotic index, epidermal regeneration has been shown to lessen with age, leaving a smaller number of keratinocytes in older subjects. Several mechanisms by which the mitotic index of keratinocytes is altered with age have been previously reported, such as decreasing levels of epidermal growth factor (Wang et al. 2020), and rising intracellular calcium (Micallef et al. 2009). Likewise, the augmentation in keratinocyte apoptosis found with aging (Wang et al. 2020) would also help explain the reduced count observed in our results.

However, paradoxically, our data showed no variation in total epidermal cellularity, although a decrease in keratinocytes was suggested. To explain this observation, we also measured other epidermal cell types (i.e., melanocytes, Langerhans cells, and Merkel cells), observing a modest increase with age. These results might account for the stable total cell count in the epidermis. Melanocytes are cells that produce melanin, the pigment that protects against UV rays (Brenner and Hearing 2008). However, although these increase with age, this does not imply greater photoprotection if one takes into account the alterations in its functions (including impaired melanosome transport or glucose metabolism) demonstrated in some studies (Park et al. 2023). Contrariwise, Langerhans cells are cutaneous dendritic cells that exert immunological protection, being mainly antigen presenters (Romani et al. 2003). These are also elevated in the oldest group, probably due to immune system deregulation in these older individuals (Fülöp et al. 2019; Pawelec 2018). Lastly, although no significant changes were found in Merkel cells (sensory neuroendocrine cells), our results are in line with those reported by Moll et al. (1984), who observed these cells in high concentration at the fetal level, they were decreased during childhood, and in older ages their production may increase again, probably due to greater exposure to harmful stimuli (Wright et al. 2017).

Taken altogether, these findings suggest that the decrease in epidermal thickness caused by intrinsic aging most likely results from a loss in cellular turgor of keratinocytes (which become shorter and wider; Farage et al. 2013). This process is due to dehydration, mainly of the stratum corneum, because of alterations in binding proteins and lipid structures (Waller and Maibach 2006; Rogers et al. 1996). Dehydration would also contribute to maximize epidermal inflammation (Wang et al. 2020), which further supports the results obtained in this field.

### Effect of intrinsic aging in dermis composition

Our results from dermal layer analysis highlighted an increase in papillary dermis and a reduction in reticular dermis thickness with aging. To elucidate this, the area occupied by GAG was first quantified. According to our data, intrinsic aging influences the presence of GAG, as reflected by an augmentation in the papillary layer and a reduction in reticular dermis. Admittedly, these results are controversial in comparison with the current literature. Some studies have suggested that intrinsic skin aging induces overexpression of GAG (Timár et al. 2000), while others have reported that photoaging, but not intrinsic aging, provoked increased GAG expression (Waller and Maibach 2006). Further research has uncovered that certain key GAG skin components, like decorin proteoglycan, undergo a reduction in size, leading to a decrease in dermal volume occupied by GAG (Li et al. 2013). Moreover, the increased papillary dermis can be viewed as an indirect consequence of the reduced number and length of ridges and papillae, as a result of which this space becomes occupied by the papillary dermis.

Since fibers are also components of dermal interstitium, modifications in their presence were also determined in subjects at different ages. A previous study from our group reported that the area of the papillary dermis occupied by collagen fibers and the thickness of its bundles were reduced (Marcos–Garcés et al. 2014). In terms of elastic fibers, although some studies suggest no differences in the density of elastic fibers due to intrinsic aging (Timár et al. 2000), our data indicates that the number of elastic fibers within reticular dermis is progressively reduced, especially when reaching adulthood. Contrasting our results with those of other studies, it seems that cutaneous elastin undergoes a series of changes in composition and structure over time (Pasquali-Ronchetti and Baccarani-Contri 1997), while elastic fibers show tortuosity and distortion which cause loss of elasticity (Imayama and Braverman 1989). Thus, unlike the impact of photoaging, which produces an increase in elastic fiber density (Hunzelmann et al. 2001), intrinsic aging therefore leads to progressive degradation (Vitellaro–Zuccarello et al., 1994) as well as cumulative damage in these fibers (Waller and Maibach 2006). In addition, alteration of skin elasticity with age may result from fibroblasts loss (Gunin et al. 2014), reduced biosynthetic activity, and variations in extracellular matrix macromolecules (Frances et al. 1990).

A reduction in dermal cellularity was noted in both the papillary and reticular layers, thus lending credence to the assertion that fibroblast proliferation is decreased (Gunin et al. 2011). Initially, the possibility of an increase in the inflammatory cell population had been suggested (Lee et al. 2021), since other studies have shown a gradual increase in both mast cells and CD45^+^ cells (Gunin et al. 2011). However, our results regarding mast cell density displayed no changes affecting the older group.

Finally, skin appendages (hair follicles and sweat glands) are notably reduced from youth onwards (Kamberov et al. 2015), which could be explained by a reduction in fibroblast number, circulating growth factors levels, and microvessels density. To corroborate this hypothesis, we subsequently analyzed changes in the number of capillaries at different age stages. According to our data, the number of dermal vessels peaks in children (0–12 years), diminishing notably in group 4 (≥ 55). These results are in line with a previous study demonstrating that a decrease in dermal vascular density is related to downregulation of the vascular endothelial growth factor signaling cascade and lower levels of von Willebrand factor (Gunin et al. 2014). Despite several studies that agree on the reduction of cutaneous blood flow (Waller and Maibach 2005), this finding is a bit controversial as other studies support just the opposite (Chung et al. 2002). Nonetheless, this reduction could explain numerous skin changes attributed to intrinsic aging, being partly a cause and consequence of atrophy, for example, of the appendages.

## Conclusions

Intrinsic aging has a dramatic effect on skin composition, as reflected by a decay in epidermal thickness and a flattened dermo-epidermal junction. The papillary dermis becomes wider and contains a larger GAG concentration, while exactly the opposite evolution is observed in the reticular layer, together with a decline in the quantity of elastic fibers. Both dermal regions experience a drop in vascularization, cellularity, and appendages. This study establishes the basis of skin chronological evolution, highlighting the need for further research on the molecular mechanisms responsible for intrinsic aging and potential targets of antiaging strategies.

## Data Availability

Data will be made available on request.
